# Antimicrobial resistance in human and animal pathogens in Zambia, Democratic Republic of Congo, Mozambique and Tanzania: an urgent need of a sustainable surveillance system

**DOI:** 10.1186/1476-0711-12-28

**Published:** 2013-10-12

**Authors:** Stephen E Mshana, Mecky Matee, Mark Rweyemamu

**Affiliations:** 1 Department of Microbiology/Immunology Weill Bugando School of Medicine, CUHAS-Bugando, Mwanza, Tanzania; 2 Department of Microbiology/Immunology, Muhimbili University of Health and Allied Sciences, Dar es Salaam, Tanzania; 3 Southern African Centre for Infectious Disease Surveillance, Sokoine University of Agriculture, Morogoro, Tanzania

## Abstract

A review of the published and unpublished literature on bacterial resistance in human and animals was performed. Sixty-eight articles/reports from the Democratic Republic of Congo (DRC), Mozambique, Tanzania and Zambia were reviewed. The majority of these articles were from Tanzania. There is an increasing trend in the incidence of antibiotic resistance; of major concern is the increase in multidrug- resistant *Escherichia coli*, *Klebsiella pneumoniae*, *Staphylococcus aureus*, *Vibrio cholera*, non-typhoid *Salmonella* and other pathogens responsible for nosocomial infections. The increase in methicillin- resistant *Staphylococcus aureus* and extended-spectrum beta-lactamase (ESBL) producers in the countries under review confirms the spread of these clones worldwide. Clinical microbiology services in these countries need to be strengthened in order to allow a coordinated surveillance for antimicrobial resistance and provide data for local treatment guidelines and for national policies to control antimicrobial resistance. While the present study does not provide conclusive evidence to associate the increasing trend in antibiotic resistance in humans with the use of antibiotics in animals, either as feed additives or veterinary prescription, we strongly recommend a one-health approach of systematic surveillance across the public and animal health sectors, as well as the adherence to the FAO (Food and Agriculture Organization)-OIE (World Organization of animal Health) –WHO(World Health Organization) recommendations for non-human antimicrobial usage.

## Introduction

The introduction of penicillin, in the early 1940s, was perceived as marking the end of infectious diseases [1]. However, the emergence of resistant strains was reported just a few years after its use. Since then, resistant clones to various classes of antibiotics have been found to spread worldwide [2]. In some areas, more than 90% resistance has been reported to commonly used antibiotics such as penicillin, ampicillin, co-trimoxazole and gentamicin [3]. The overuse of antibiotics in human and animals has contributed to the emergence of resistant clones [4,5]. It is a fact that the availability of antimicrobials and their proper use have reduced morbidity and mortality due to infectious disease [http://www.cdc.gov/drugresistance/index.html]. In developed countries, the use of antibiotics is strictly controlled, which is not the case in developing countries. The treatment of bacterial infections in Africa is largely empirical and in most instances, there are no laboratory results to guide therapy. Moreover, there are no data on common bacterial isolates and their susceptibility patterns from larger surveillance studies aimed at developing tools for therapeutic guidance. Developing countries bear 95% of the global infectious diseases burden and rely on empirical antimicrobial treatment to counteract these diseases [6]. This has resulted in many infectious diseases, once easily curable, to become untreatable [7-9].

The burden of antimicrobial resistance (AMR) is rapidly growing across antibiotic classes. The emergence of methicillin- resistant *Staphylococcus aureus* (MRSA) clones spreading among animals and human has made AMR an issue of public health importance [10]. Recently, the emergence of NDM-1 has made infection due to multi-resistant gram negative bacteria untreatable, especially in developing countries where there is no alternative treatment available [11]. Unfortunately, coordinated surveillance of the clones involved is lacking, especially in developing countries in Africa.

This review was undertaken to summarize the patterns and trends of resistance to commonly used antibiotics among common bacterial isolates from humans and animals from 1990s to 2012 in Democratic Republic of Congo (DRC), Mozambique, Tanzania and Zambia. The data from this review will be used to provide recommendations on priority research area that could address the development and spread of antibiotic resistance in humans and animals.

## Methods

In this review, literature from four different countries is appraised and reported. Though these countries present differences in terms of local agriculture, economy, state of health care services, political situation, etc., they all have no clear policy on antibiotic use hence at risk of increased antibiotic resistance. Also, these countries have all introduced the one-health concept under the South African Centre of Infectious Diseases (SACIDS) aiming at providing data for evidence based management of infectious diseases.

### Study design and search strategy

A systematic literature review was conducted for original articles on bacterial isolates, resistance patterns in humans and animals from DRC, Mozambique, Tanzania and Zambia, published from 1990 to the end of July 2012. The study focused on all bacterial pathogens with the exception of *Mycobacterium tuberculosis*. A systematic search of online databases including PubMed/Medline, Embase, Popline, Global Health, Google and Web of Knowledge was undertaken. We used the search terms “bacterial isolate resistance patterns”, “antibiotic use”, “antimicrobial resistance”, “microbial resistance”, “susceptibility”, “resistance surveillance” in “human or animal use” combined with the name of the different countries of interest. References of all articles were searched to identify further articles. Articles were reviewed and publications using original data on resistance in animals and humans were included. The studies’ design, setting, demographic data and microbiological methods were appraised. All studies involved human were approved by ethics committee and informed consent obtained. We also consulted the WHO, OIE and FAO websites for relevant publications. New links displayed beside the abstracts were followed and retrieved.

## Results

### Overview of study design and microbiological susceptibility methods

A total of 68 articles published between 1990 and 2012 describing bacterial pathogens and/or antibiotic resistance in humans and animals were retrieved; all articles were included in this review. Of 68 articles 40 (59%) reported data on the susceptibility pattern; of these 88% used disc diffusion method (Additional file 1: Table S1). The CLSI guideline was used in 60% of articles, 3 articles used French Society for Microbiology and 1 article used European Committee of Antimicrobial and Susceptibility Testing (EUCAST) and 12 articles did not state the guideline used. Thirteen (33%) articles did not report the use of control strains. The majority of the studies were short-term clinical studies and retrospective clinical studies while 4 were surveillance studies (3 from Tanzania and 1 from Mozambique). The distribution of articles by country is seen in Table 1. No articles describing antibiotic resistance in animals were found from DRC and Mozambique. Most of the human studies from DRC, Mozambique, Tanzania and Zambia were carried out in urban settings in tertiary hospitals, with only one study from Tanzania involving primary health facilities, while 3/11 (27%) of human studies from Mozambique involved children from a rural hospital.

**Table 1:** Articles distribution per country and source of samples

***Country***	***Human***	***Animal***
**Tanzania**	33	3
**Zambia**	6	5
**DRC**	9	1
**Mozambique**	11	0
**Total**	**59**	**9**

### *Escherichia coli* and *Klebsiella pneumoniae* causing Urinary tract infections (UTI) and blood stream infections

*Escherichia coli* and *Klebsiella pneumoniae* have been identified as the most common causes of Urinary Tract Infection (UTI) in Tanzania; contributed 47% of all UTI. Ten studies from Tanzania reported on UTI and the majority of them (9/10) were short clinical studies involving pregnant women and children [12-21]. No data regarding these species on UTI were found from DRC, Mozambique and Zambia. Resistance rates in different studies are seen in Table 2; *Klebsiella* spp showed higher resistance rates than *Escherichia coli*. Among *Escherichia coli* mean resistance ranges from 14% for ciprofloxacin to 85% for ampicillin while among *Klebsiella* spp mean resistance ranges from 20% for nitrofurantoin to 85% for ampicillin. There is an increased trend of these isolates to become resistant to commonly used antibiotics. Among *Escherichia coli* isolates from urine, ampicillin resistance has been found to increase from 17% in 1995–1996 [20] to more than 93% in 2009 [12,19]. In addition, an increasing resistance trend to gentamicin among *Escherichia coli* isolated from urine specimens has been observed; the rate of gentamicin resistance in Tanzania was found to range from 7% at the Muhimbili National hospital (MNH) in 2003 to more than 44% in the same hospital in 2011 [15,20].

**Table 2:** **Antimicrobial resistance rates of *Escherichia coli *and *Klebsiella pneumoniae *from Urine Tanzania** (**ref**: [12-15,19-21])

Antibiotic(N*)	**Resistance rates in different studies%*Escherichia coli*(range)**	Mean resistance n/N* (%)
Ampicillin (518)	92, 96, 53, 98, 100, 69, 80 **(53–100)**	438/518 (85)
Augmentin (501)	53, 70, 88, 85, 37 **(37–88)**	337/501 (67)
Gentamicin (518)	7, 38, 6, 23, 32, 44 **(6–44)**	130/518 (25)
Co-trimoxazole (339)	80, 65, 95,97, 50 **(50–97)**	262/339 (77)
Ciprofloxacin (501)	8, 30, 12, 9, 13 **(8–30)**	72/501 (14)
Ceftriaxone (339)	51, 29, 14, 27, 19 **(14–51)**	95/339 (28)
Ceftazidime (306)	50, 11, 14 **(11–50)**	77/306 (25)
Nitrofurantoin (289)	23, 6, 13, 31**(6–31)**	53/289 (18)
	Resistance rates in different studies *Klebsiella pneumoniae* % **(range)**	
Ampicillin (193)	56, 100, 98 **(56–100)**	163/193(85)
Augmentin (193)	11, 78, 86 **(11–86)**	112/193(58)
Gentamicin (193)	11, 28, 38 **(11–38)**	50/193 (26)
Co-trimoxazole (193)	56, 83, 95 **(95–56)**	150/193 (78)
Ciprofloxacin (193)	19, 19, 44 **(19–44)**	53/193 (27)
Ceftriaxone (193)	33, 66, 46 **(33–66)**	93/193 (48)
Ceftazidime (52)	52	27/52 (52)
Nitrofurantoin (193)	18, 21, 22 **(18–22)**	37/193 (20)

N*=Total number of isolates tested.

*Escherichia coli* and *Klebsiella pneumoniae* also have been found to cause blood stream infections, especially in neonates [22-26]. *Escherichia coli* was the most common isolate in blood stream community- acquired infections while *Klebsiella spp* was the commonest isolate in health care associated infection [22-24]. Mean resistance rates of *Escherichia coli* from the blood were 2%, 15%, 28%, 37%, 38%, 55%, 57%, 76% and 95% for meropenem, ciprofloxacin, ceftazidime, gentamicin, ceftriaxone, augmentin, tetracycline, co-trimoxazole and ampicillin respectively (Table 3). With exception for meropenem, and ciprofloxacin *Klebsiella* spp had higher resistance rates than *Escherichia coli*. *Escherichia coli* and *Klebsiella pneumoniae* strains from the blood displayed higher rates of resistance than those from urine specimens against common antibiotics such as ampicillin, co-trimoxazole and gentamicin [22,24,25]. In this review resistance to third generation cephalosporins was observed in more than 20% of hospital-acquired *Klebsiella* spp and *Escherichia coli*.

**Table 3:** **Antimicrobial resistance rates of *Escherichia coli *and *Klebsiella pneumoniae *from the blood**, **Mozambique and Tanzania** (**ref**: [22-26])

**Antibiotic**	**Resistance rate in different studies%*Escherichia coli*(range)**	Mean resistance n/N* (%)
Ampicillin	84, 96 , 85, 100, 96 **(84–100)**	227/240 (95)
Augmentin	40, 25, 69, 86 **(25–86)**	47/86 (55)
Tetracycline	55, 59 **(55–59)**	28/49(57)
Gentamicin	13, 29, 46, 68, 28 **(13–68)**	87/234 (37)
Co-trimoxazole	72, 87, 54, 77, 90 **(54–90)**	173/227 (76)
Ciprofloxacin	40, 8, 8, 4 **(4–40)**	13/86 (15)
Ceftriaxone	12, 54, 50 **(12–54)**	23/59(38)
Ceftazidime	0.0, 12, 50, 50 **(0–50)**	24/86 (28)
Meropenem	0.0, 0.0, 4 **(0–4)**	1/59 (2)
	Resistance rate in different studies % *Klebsiella pneumoniae***(range)**	
Ampicillin	91, 100, 100, 100, 100 **(91–100)**	202/206 (98)
Augmentin	32, 47, 38, 84 **(32–84)**	97/194 ( 50)
Tetracycline	67, 62 **(62–67)**	91/141(65)
Gentamicin	40, 47, 47, 67, 18 **(18–67)**	91/206 (44)
Co-trimoxazole	63, 63, 94, 79, 70 **(64–94)**	151/206 (74)
Ciprofloxacin	0.00, 0.00, 0.00, 8 **(0–8)**	4/194 (2)
Ceftriaxone	50	25/50 (50)
Ceftazidime	6, 21, 15, 49 **(6–49)**	42/184 (22)
Meropenem	0.00, 0.00, 2 **(0–2)**	1/160 (1)

N*=Total number of isolates tested.

One study in Tanzania reported data from surveillance study [24]. In that study a total of 1936 and 1771 of *E*.*coli* and *Klebsiella pneumoniae* were isolated in 2 year period from 1^st^ January 1998 to 31^st^ December 1999. Majority of these isolates were from urine specimens, others were from pus, blood and other routine specimens. *Escherichia coli* were 80%, 28%, 5%, 77%, 8%, 76%, 32% and 13% resistant to ampicillin, amoxicillin/clavulanic acid, ceftazidime, tetracycline, gentamicin, co-trimoxazole, nitrofurantoin and quinolones while the rates of resistance to similar antibiotics for *Klebsiella pneumoniae* were 85%, 32%, 6%,66%, 14%, 69%, 53% and 6% respectively.

### Extended –spectrum beta-lactamase (ESBL) producing *Escherichia coli* and *Klebsiella pneumoniae*

Data on ESBL producing *E*. *coli* and *Klebsiella pneumoniae* are limited in all the countries under review. Of 302 *E*. *coli* tested for ESBL production in these countries 88/302 (29%) were found to be ESBL producers while of 283 *Klebsiella pneumoniae* tested 116/282 (41%) were ESBL producers [3,19,27-30]. Different ESBL alleles have been reported including CTX-M-15 *Escherichia coli* ST 131 which has been reported worldwide [28]. Other reported alleles include TEM-63, SHV-2a and SHV-12 [29].

### Blood stream infection due to *Salmonella* spp

There is increase trend of *Salmonella* spp causing blood stream infection in developing countries especially among HIV/AIDS patients. In these countries most of *Salmonella* spp from the blood were found to be resistant to ampicillin, co-trimoxazole and chloramphenicol (Table 4) while being sensitive to ciprofloxacin [26,31-37]. An increased resistance trend of *Salmonella* spp to ciprofloxacin in DRC from 0.0% in 2009 to 15.4% in 2012 was noted [32,33].

**Table 4:** **Antimicrobial resistance rates of *Salmonella *spp from the blood**, **Zambia**, **DRC**, **Mozambique and Tanzania ref**: [26,32,33,37]

**Antibiotic**	**Resistance rates in different studies%(range)**	Mean resistance n/N* (%)
Ampicillin	100, 65, 74, 69 **(65–100)**	477/620(77)
Co-trimoxazole	100, 58, 66, 38 **(38–100)**	404/620 (65)
Chloramphenicol	100, 40, 55, 85 **(40–100)**	434/620 (70)
Gentamicin	16, 23 **(16–23)**	78/400(20)
Ciprofloxacin	0, 8, 15 **(0–15)**	32/225 (14)

### *Salmonella* spp, *Shigella* spp, *Vibrio cholera*, *Campylobacter* spp and diarrheagenic *E*.*coli*; and their susceptibility patterns

Diarrheal diseases account for approximately 25% of all deaths in <5 year-old children in developing countries [38,39] and common pathogens (*Campylobacter* spp, *Salmonella* spp, *Shigella* spp and Diarrheagenic *E*. *coli*) causing diarrhea in human have been found in animals. Except for erythromycin resistance rates to ampicillin, co-trimoxazole, chloramphenicol and tetracycline are higher among *Shigella* spp than *Salmonella* spp in these countries (Table 5) [40-45]. *Shigella* spp have been found to be common in DRC and it was noted that more than 50% of *Shigella* spp from that country were multi-drug resistant strains. This is a 4-fold increase compared to a study which was done in 1964 which demonstrated resistance to two antibiotics to be 10% [42,45].

**Table 5:** **Antimicrobial resistance rates of *Salmonella *spp**, ***Shigella *spp and diarrheagenic *E***. ***coli *from the stool**, **Zambia**, **DRC**, **Mozambique and Tanzania**

**Antibiotic**	**Resistance rate in different studies%*Salmonella*spp(range):ref:[**[41,42]**]**	Mean resistance n/N* (%)
Ampicillin	25	10/40(25)
Co-trimoxazole	78, 18 **(18–78)**	29/61(48)
Chloramphenicol	15	6/40 (15)
Tetracycline	94, 15 **(15–94)**	33/61(55)
Cefotaxime	14	3/21(14)
Erythromycin	86	18/21(86)
	Resistance rate in different studies % *Shigella* spp **(range)**: ref: [43-45]	
Ampicillin	97, 56 **(56–97)**	130/171 (77)
Co-trimoxazole	97, 84 **(84–97)**	155/171 (91)
Chloramphenicol	94, 52 **(52–94)**	125/171(73)
Tetracycline	98, 66 **(66–98)**	140/171 (82)
Erythromycin	19	12/62 (19)
	Resistance rate in different studies % diarrheagenic *E*. *coli***(range)**: ref [51,52,54]	
Ampicillin	72, 100 **(72–100)**	89/104 (86)
Co-trimoxazole	71, 60, 58 **(58–71)**	74/118 (63)
Chloramphenicol	45, 100 **(45–100)**	75/104 (73)
Tetracycline	48, 100 **(48–100)**	77.104 (74)
Erythromycin	83	12/14 (83)
Ciprofloxacin	1	1/94 (1)
Nitrofurantoin	23	3/14 (23)

N*=Total number of isolates tested.

**Figure 1:**
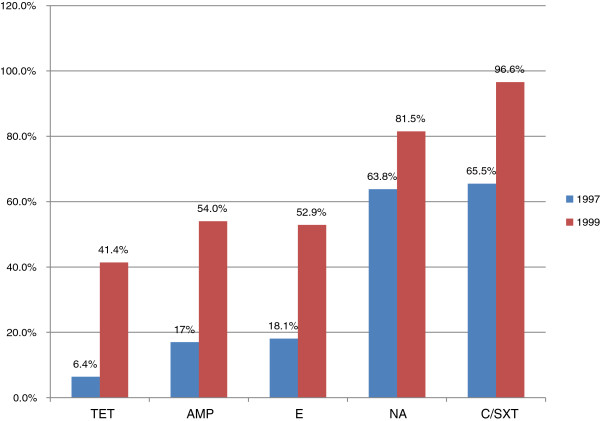
**Pattern of resistance of Vibrio cholerae isolates from Tanzania to tetracycline (TET), erythromycin (E), nalidixic acid (NA) and chloramphenicol / co**-**trimoxazole in 1997 and 1999.**

Five studies [46-50] reported on *Vibrio cholera* with four of them reporting on the susceptibility pattern of *Vibrio cholera* among isolates obtained during outbreaks [47-50]. In Zambia [47], *Vibrio cholera* 01 strains isolated from first two outbreaks (1990–1991), showed a low level resistance (2-3%) to tetracycline. The use of tetracycline caused the resistance to increase to 95% in the subsequent outbreaks in 1992. Similar observations were reported in Tanzania [48], whereby it was noted that there was an increase in resistance patterns against *V*. *cholera* between outbreaks occurred in 1997 and later outbreaks in1999 (Figure 1). In Mozambique; of 175 rectal swabs or stool samples from patients with diarrhea, 58 strains of *V*. *cholerae* 01 were isolated between January 7 and March 8, 2004. The antimicrobial susceptibility of 15 isolates showed that all strains were sensitive to tetracycline, ampicillin, furazolidine, erythromycin and ciprofloxacin but resistant to co-trimoxazole [49].

Five studies [51-55] investigated the presence diarrheagenic *E*. *coli* strains in these countries with 3 of studies [51,52,54] describing susceptibility pattern to various antibiotics. The mean resistance rates of diarrheagenic *E*. *coli* isolated in these countries for ciprofloxacin, nitrofurantoin, co-trimoxazole, chloramphenicol, tetracycline, erythromycin and ampicillin are seen in Table 5.

*Campylobacter* spp is one the commonest causes of diarrhea in children but only four short-term studies could be found on the subject from Tanzania and Mozambique [44,56-58]. The prevalence of campylobacter infection in human in Tanzania was found to range from 2.6% to 9.3% [56-58]. All these human studies from Tanzania did not test for the susceptibility pattern of the isolates. The other study [59] in the same area investigated the presence of *Campylobacter* spp in domestic ducks; found that 80% of ducks had thermophilic campylobacter infection. Of 50 isolates tested for susceptibility pattern 48%, 74% and 82% were resistant to cefuroxime, tetracycline and amoxycillin respectively while 35% of the isolates were resistant to erythromycin. The resistance rates to norfloxacin and ciprofloxacin were 10% and 16% respectively (Table 6). In Mozambique of 529 samples from children 9 (1.7%) had campylobacter infection, all nine *Campylobacter* spp strains isolated from children with diarrhea were susceptible to erythromycin while 1 (11) was resistant to ciprofloxacin [44].

**Table 6:** **Antimicrobial resistance rates of *Escherichia coli *from animals and *Campylobacter *spp in Mozambique**, **Tanzania and Zambia** (**ref**: [51,59,74,77])

**Antibiotic**	**Resistance rate in different studies**: ***Escherichia coli*%(range)**	Mean resistance n/N* (%)
Ampicillin	80, 90, 88 **(80–90)**	356/414 (86)
Augmentin	82, 74, 83 **(74–83)**	276/346 (79)
Gentamicin	63, 67 **(63–67)**	191/294(65)
Co-trimoxazole	36, 71, 91 **(36–91)**	282/427 (66)
Tetracycline	4, 96 **(4–96)**	154/307 (50)
	*Resistance rates Campylobacter jejuni %***(range)**	
Amoxicillin	82	41/50 (82)
Tetracycline	74, 22 **(22–74)**	28/59 (48)
Erythromycin	35, 0.0 **(0–35)**	18/59 (30)
Cefuroxime	48	24/50 (48)
Norfloxacin	10	5/50 (10)
Ciprofloxacin	16, 11 **(11–16)**	9/59 (15)

N*=Total number of isolates tested.

### Methicillin-resistant *Staphylococcus aureus* (MRSA) and Health care-associated infections (HCAIs)

The WHO estimates that health care-associated infections (HCAIs) in developing countries are found in 15.5% of admissions, which is 3 times higher than the rate in developed countries [60,61]. One form of infection, the surgical site infection, is particularly common in developing countries due to the economic constraints of hospitals and lack of hygiene, infection prevention strategies and staff [29,62-66]. *Staphylococcus aureus* has been found to be the predominant pathogen causing SSI in these countries with 13% of the bacteria exhibiting MRSA phenotype (Table 7). Interestingly, the prevalence of MRSA in Tanzania has been increasing, at the Muhimbili National Hospital, the prevalence was 0.4% in 1999 [66], 2% in 2004 [24], and 23.3% in 2010 [25]. At the Bugando Medical Center (BMC), the prevalence of MRSA was 16.3% [67] in 2009 and 18.8% in 2011 [61].

**Table 7:** **Antimicrobial resistance rates of *Staphylococcus aureus ***, **Zambia**, **DRC**, **Mozambique and Tanzania** (**ref**: [26,62,68-72])

**Antibiotic**	**Resistance rate in different studies%(range)**	Mean resistance n/N* (%)
Penicillin	90, 90, 85 **(85–90)**	291/329 (88)
Erythromycin	35, 5, 7 **(5–35)**	51/327 (16)
Clindamycin	22	10/46 (22)
Co-trimoxazole	31, 73 **(31–73)**	145/278 (52)
Chloramphenicol	37, 7 **(7–37)**	63/287 (22)
Gentamicin	5	9/182 (5)
Tetracycline	48, 28 **(28–48)**	56/147 (38)
MRSA	31, 16, 2, 27, 2, 8 **(2–31)**	317/2472 (13)

N*=Total number of isolates tested.

Nine studies [26,62,63,67-72] reported the resistance patterns of *Staphylococcus aureu*s with only two studies providing molecular insights of MRSA. The mean resistance rates ranges from 5% to 88% to various antibiotics as seen in Table 7. Regarding spa types, sixteen different spa types (t012, t021, t122, t186, t279, t701, t1855, t1877, t224, t084, t304, t934, t1247, t2864, t7722 and t7723) were observed in one study in Zambia [69], while in Tanzania; the new MRSA clone ST1797/t7231 has been isolated thus emphasizing the diversity of MRSA clones in Africa [72]. In Mozambique; of 24 MRSA isolates 6(25%), 3(12.5%) and 1(4.2%) were also resistant to tetracycline, erythromycin and co-trimoxazole and only 1 strain was resistant to all antibiotics tested [26].

### Antibiotic resistance in isolates from animals

Eight reviewed studies [51,59,73-78] investigated the magnitude of bacterial diseases in animals with only 4 studies [51,59,77,78] reporting resistance profile to various antibiotics. As for *Escherichia coli* from human; *Escherichia coli* from animals exhibited high resistance rates ranging from 50% for tetracycline to 86% for ampicillin (Table 7). *Salmonella enteritidis* was found to contaminate 3.8% and 4.7% of eggs and chicken carcasses respectively. All *Salmonella enteritidis* isolates were found to be sensitive to gentamicin, ampicillin, tetracycline, co-trimoxazole, amoxicillin, furazolidine and chloramphenicol.

## Discussion

In the developing countries under review, there are limited data from large surveillance studies on antimicrobial resistance. In addition few short clinical studies document the susceptibility pattern of common pathogens from human and animals. This may partly be due to the lack of microbiological facilities in many health facilities in developing countries [79]. Our findings emphasize the need for coordinated efforts to improve the diagnosis of infectious diseases in developing countries coupled with surveillance of antimicrobial resistance in these countries. Together with the increased effort by WHO to control malaria transmission, other potential causes of fever should be taken into consideration; and appropriate antibiotic treatment will reduce morbidity and mortality resulting from other causes of fever.

Despite few studies blood stream and urinary tract infections have been found to be common in these countries as demonstrated in this review and it should be noted that the endemicity of HIV has changed their epidemiology in Africa. Apart from *Salmonella* spp; multi-drug *Escherichia coli* and *Klebsiella pneumoniae* were found to be common causes of blood stream infections and UTI. Increased trend of these isolates to become resistant to ampicillin, gentamicin and third generations’ cephalosporins was noted; this could be due to overuse of these drugs in the community and hospitals, in all countries reviewed no clear antibiotic policy was found.

ESBL has been found to be a threat, especially as a cause of nosocomial infections. Prevalence as high as 50% have been observed among *Klebsiella pneumoniae* from inpatients in these countries. The occurrence of the *Escherichia coli* clone ST 131 in Tanzania confirms that resistant clones can spread from one part of the world to another [28]. Low mean resistance rates to meropenem were observed in these countries; this could be explained by the fact that this drug is expensive and not available in the market. There is an urgent need of antibiotic policy in these countries because in countries where carbapenems have been misused, such as India or Pakistan, outbreaks of carbapenems resistant *Escherichia coli* and *Klebsiella pneumoniae* have been experienced [11]. Recently, the emergence of NDM-1 plasmid mediated carbapenems resistance has been noted, spreading from India to Europe, USA and Africa. Joint efforts are needed to control the spread of NDM-1.

In this review few studies were found to address enteric pathogens, it was noted that *Shigella* spp were more resistance than *Salmonella* spp; an increase trend of multi--drug resistant *Shigella* spp in DRC was noted. Similarly most of diarrheagenic *Escherichia coli* were resistant to commonly used antibiotics (ampicillin, co-trimoxazole, tetracycline and erythromycin). This could be due to self prescription of these antibiotics for the treatment of diarrhea episodes as evidenced by increased resistance of *Vibrio cholerae* strains between outbreaks in Zambia and Tanzania. In all countries under review, many patients buy antibiotics from private pharmacies and drug shops for self-medication before seeking medical professional care [80-82]. In addition *Campylobacter* spp were found to be resistant to ciprofloxacin, cefuroxime and erythromycin. This situation needs to be further investigated with standardize microbiological method so that the real magnitude can be established or confirmed.

In addition, MRSA appears to be an emerging problem; the problem might be underestimated because not all laboratories in these countries are performing MRSA identification. However *Staphylococcus aureus* has been the major cause of SSI, as documented in few studies from these countries. The increase trend of MRSA as noted in Tanzania; necessitate the coordinated surveillance to determine the evolution of these strains in Africa. MRSA isolates have been isolated from animals and we need, therefore, to compare the genotypes between animals and humans, as evidenced by the diversity of MRSA genotypes in Tanzania and Zambia [68-70].

Few laboratories are routinely conducting testing for ESBL and MRSA detection. This observation stresses the need for governmental and non- governmental organizations to provide sustainable support to improve laboratory capacity in developing countries. This should go hand in hand with the establishment of a quality assurance system to ensure quality microbiological results from all laboratories. As noted in Tanzania, there is an increased resistance trend to ceftriaxone and ceftazidime among isolates causing nosocomial infections. Improving diagnostic facilities and research capacity to determine the evolution of ESBL and MRSA clones in Africa is no longer an option but is mandatory, especially when the treatment of ESBL producing isolates and MRSA becomes too expensive for countries like DRC, Mozambique, Tanzania and Zambia.

Unauthorized use of antibiotics seems to be common, both in medical and veterinary settings. While we have found no evidence linking antimicrobial resistance in human cases with the use of similar antibiotics in animals, there is a need for a coordinated one health based surveillance of the antimicrobial resistance in humans and animals. It appears also relevant to follow the recommendations of the Joint FAO-OIE-WHO on non-human antimicrobial usage.

## Conclusions

Relevant information on bacterial diseases is limited in DRC, Mozambique, Tanzania and Zambia. Moreover, there are no national policies guiding surveillance. Lack of detailed microbiological method was noted in various studies with no detailed information regarding quality assurance; but despite this limitation an increased trend of resistance to commonly used antibiotics such as ampicillin, co-trimoxazole, gentamicin, erythromycin, tetracycline and third generation cephalosporins was noted. This might be exacerbated by behavioral factors, incentive motivate prescribing, and the dispensing and purchasing of antibiotics when they are inappropriate for treating a specific condition. Other contributing factors include the drugs’ quality or the use of partial doses, and patients’ demand for symptomatic eradications. A systematic national surveillance system is urgently needed to provide data on the levels of bacterial diseases and drug resistance in common pathogens from hospitals and communities. In addition, a comparative molecular epidemiology study to compare human and animals isolates is urgently needed to shade a light of the transmission of bacterial pathogens, especially at the community level. The rising incidence of MRSA and ESBL infections points to the need to include the heightened the regimes for hospital facility cleanliness as part of the policy for the use of antibiotics and antibiotic stewardship in order to minimize the risk of hospital-acquired infections with MRSA and ESBL producing isolates.

## Competing interests

The authors declare that they have no competing interests.

## Authors’ contributions

SEM, MM and MR participated in the literature search; SEM prepared the first draft of the manuscript. All authors read and approved the final manuscript.

## Supplementary Material

Additional file 1: Table S1 Showing Methodology used for susceptibility testing, population sampled and specimens collected in various studies. Click here for file
