# Supplementary material for: Antimicrobial resistance in human and animal pathogens in Zambia, Democratic Republic of Congo, Mozambique and Tanzania: an urgent need of a sustainable surveillance system
Source: Ann Clin Microbiol Antimicrob. 2013 Oct 12;12:28. doi: 10.1186/1476-0711-12-28 (PMC3852305; doi:10.1186/1476-0711-12-28)
Supplement: Additional file 1: Table S1 — Showing Methodology used for susceptibility testing, population sampled and specimens collected in various studies. [file 1476-0711-12-28-S1.pdf]

Supplementary Table 1: Showing Methodology used for susceptibility testing, population sampled and specimens collected in various studies

Ref	Authors	Population	Specimens	Community/Hospital	Method of susceptibility	Guideline	Control Strains
12	Festo et al	Children 370	Urine	Hospital	Disc diffusion	CLSI	Stated
13	Msaki et al	Children 231	Urine	Community/ Outpatients	Disc diffusion	CLSI	Stated
14	Massinde et al	Pregnant women	Urine	Hospital	Disc diffusion	CLSI	Stated
15	Lyamuya et al	Diabetic women	Urine	Hospital	Disc diffusion	CLSI	Stated
17	Moyo et al	Children 153, Adults 117	Urine	82 outpatients, 188 inpatients	Disc diffusion	CLSI	Stated
20	Rimoy et al	Adults and children	Urine	126 outpatients, 274 inpatients	Disc diffusion	CLSI	Not stated
21	Bloomberg et al	Pregnant women 5153	Urine	*Outpatients	Disc diffusion	CLSI	Stated
22	Bloomberg	1798 children	Blood	*Hospital	Disc diffusion	CLSI	Stated
23	Kayange et al	300 neonates	Blood	Hospital	Disc diffusion	CLSI	Stated
24	Bloomberg et al	Adults/children	Blood/urine/pus	*Inpatients/Outpatients; 2 year period	Disc diffusion	CLSI	Stated
25	Moyo	5151 Children, 1855 adults	Blood	Hospital	Disc diffusion	CLSI	Stated
26	Mandomando et al	Children 19896	Blood	*Hospital; 5 year period	Disc diffusion	CLSI	Stated
27	Bloomberg et al	Children 1787	Blood	Community and Hospital	Disc diffusion	CLSI	Stated
32	Muyembe et al	Adult/Children	Blood	Hospitals	Disc diffusion	Not stated	Not stated
33	Lunguya et al	Adult/children	Blood	Hospital	VITEK/E-test	EUCAST	Stated
37	Meremo et al	Febrile Adults	Blood	Hospital	Disc diffusion	CLSI	Stated
40	Mwansa et al	Adults	Stool	-	Disc diffusion	Not stated	Stated
41	Sosa et al	Adults	Stool	Not stated	Not stated	Not stated	Not stated
42	Lunguya O et al	Adults/Children	Stool	Community	Disc diffusion	French society of Microbiology	Stated
43	Temu et al	Adults/Children	Stool	Inpatients/Outpatients	Disc diffusion	CLSI	Stated
44	Mandomando et al	Children	Stool	Hospital	Disc diffusion	CLSI	Stated

Supplementary Table 1: Showing Methodology used for susceptibility testing, population sampled and specimens collected in various studies

45	Cavallo et al	Adults	Stool	Camps	MIC	Not stated	Not stated
47	Mwansa et al	Adults/Children	Stool	Outbreaks	Disc diffusion	CLSI	Stated
48	Urassa et al	Adults/Children	Stool	Outbreaks	Disc diffusion	Not stated	Stated
49	Ansaruzzaman et al	Adults/children	Stool	Community	Not stated	Not stated	Not stated
51	Mubita et al	Adults/children	Stool	-	Disc diffusion	Not Stated	Not stated
52	Rajii et al	Adults/Children	Stool	Hospital/community	Disc diffusion	Not stated	Not Stated
54	Mandomando et al	Children	Stool	Community	Disc diffusion	Not Stated	Not Stated
57	Mdegela et al	Human/Chickens	Stool	Community	Disc diffusion	Not stated	Not stated
61	Mawalla et al	Adults	Pus	Hospital	Disc diffusion	CLSI	Stated
66	Urassa et al	Adults	Pus	Hospital	Disc diffusion/E-test	CLSI	Stated
67	Mshana et al	Adults	Pus	Hospital	Disc diffusion	CLSI	Stated
68	Kapatamoyo et al	Adults	Pus	Hospital	Disc diffusion	CLSI	Not stated
69	Schaumburg et al	Human/Chimpanzee	Pus/others	Community	Disc diffusion	CLSI	Stated
70	Takaisi et al	Adults	Pus	Hospital	Disc diffusion	French Society for Microbiology	Stated
71	Nyembwe et al	Adults	Pus/others	Hospital	Disc diffusion	French Society for Microbiology	Stated
59	Nonga et al	Domestic duck	Intestines	NA	Disc diffusion	Not stated	Not stated
73	Ngoma et al	Cattle/pigs		NA	MIC using gar	Not stated	Not stated
76	Hang'ombe et al	eggs	Eggs	NA	Disc diffusion	CLSI	
77	Iqbal et al	Birds	intestines	NA	Disc diffusion	CLSI	Not stated

***Surveillance studies, NA: Not applicable,**
